# Congenital Pseudoarthrosis of Tibia With Anterolateral Bowing Treated With Ilizarov Ring Fixator: A Case Report

**DOI:** 10.7759/cureus.47615

**Published:** 2023-10-24

**Authors:** Amit Kale, Vishal S Patil, Parminder Singh, Harsh Raithatha, Meet Shah, Rishabh Aggarwal

**Affiliations:** 1 Orthopedics, Dr. D. Y. Patil Medical College, Hospital and Research Centre, Pune, IND

**Keywords:** neurofibromatosis 1, india, surgery, children, complications, congenital pseudarthrosis of the tibia

## Abstract

Congenital pseudarthrosis of the tibia (CPT) is a rare, dysplastic condition that is characterized by a "false joint" in the tibia, leading to potential disability. We present a rare case report of a 12-year-old male from India with a history of neurofibromatosis type 1 (NF1) and anterolateral bowing of the tibia since birth. He sustained a tibial fracture during play. X-ray evaluation confirmed the fracture, and a clinical diagnosis of CPT was established. The treatment involved corticotomy for deformity correction and stabilization using Ilizarov's ring fixation. The procedure was successful, with post-operative radiological evaluations showing significant improvement in the center of rotation of angulation (CORA) from 60° pre-operatively to 25° post-operatively. The patient was discharged with an external fixator and after seven months, transitioned to full weight-bearing ambulation with a specialized brace. The Ilizarov procedure proved to be a safe and effective treatment for CPT, offering benefits such as limb lengthening and ankle stabilization.

## Introduction

Congenital pseudarthrosis of the tibia (CPT) is a rare condition that affects one in 140,000 to one in 150,000 [1]. The term "pseudarthrosis" refers to a "pseudo-joint" and represents a bone fracture that does not naturally heal. CPT is a dysplastic pathological condition, which can lead to disability. The tibia displays a region of segmental abnormality, leading to an anterolateral curvature of the bone. The dysplastic condition can cause non-union of the tibia, which might be spontaneous or due to minor injuries [2]. In conjunction with the tibial bowing and epiphyseal growth reduction, this can cause limb shortening [3]. Although the disease usually manifests during infancy, the bowing can rarely develop between four and 12 years [1]. The etiology of CPT is congenital. Various mechanical, vascular, and genetic hypotheses have been put forth in scientific literature. However, none offer a comprehensive understanding of CPT's pathogenesis or specific location, likely due to the disease's varied nature and its sporadic link with neurofibromatosis type 1 (NF1) [2]. While both invasive and non-invasive treatment methods are being applied, managing CPT remains challenging for orthopedic surgeons [1]. The main objective of treating CPT is to attain skeletal maturity with preserved functions and anatomic alignment [4]. The index report is a pediatric case of CPT who presented with a history of trauma and managed with corticotomy for deformity correction, followed by stabilization using Ilizarov's ring fixation.

## Case presentation

A 12-year-old male presented to the orthopedics department of Dr. D. Y. Patil Medical College with acute pain and tenderness in his left leg, for the past eight hours. The patient sustained an injury due to a trivial fall during recreational play. Radiographic evaluation by X-ray revealed a fracture in the distal third of the shaft of the left tibia. Notably, the patient had a known diagnosis of NF1, evidenced by cafe au lait macules on his back. He also had a known case of anterolateral bowing of the tibia since birth, as informed and the history given by the parents. He displayed an intellectual response appropriate for his age. On examination, he was alert and oriented and demonstrated age-appropriate cognitive responses. Peripheral pulses were palpable bilaterally, and there were no open wounds. Anterolateral bowing of the left tibia is present with a shortening of 2 cm in the left tibia. A provisional clinical diagnosis of CPT was established, after medical examination.

**Figure 1:**
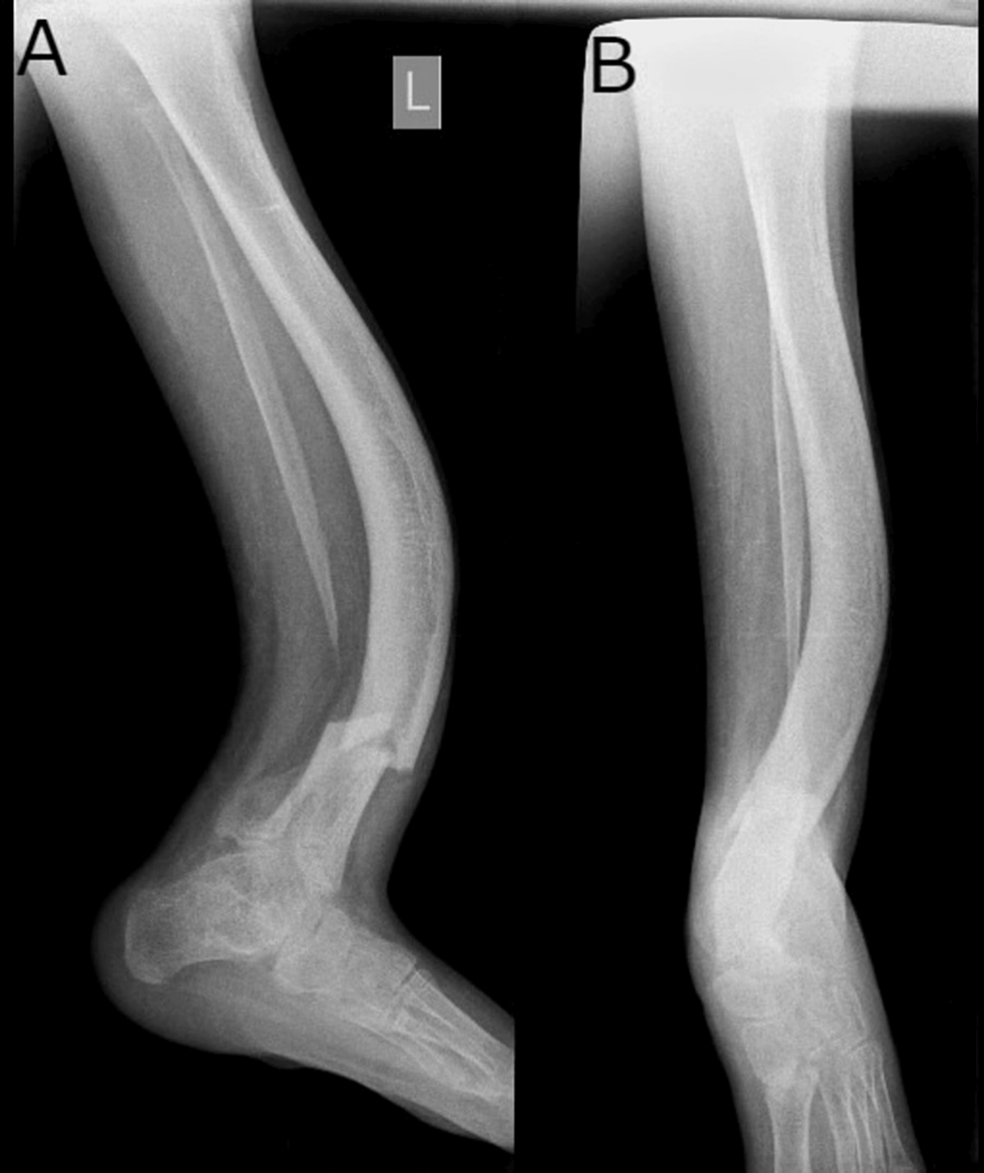
Pre-operative X-ray suggestive of distal tibia fracture with bowing. A) Lateral view and B) anteroposterior view Anterolateral bowing with a stress fracture of left distal one-third^ ^tibia pre-operative anteroposterior and lateral X-rays

X-rays, both anteroposterior and lateral views, were obtained pre-operatively (Figure 1). The patient had anterolateral bowing of the tibia with fibular hemimelia for which the patient was using clamshell orthosis for three years. The patient developed a stress fracture during recreational play below the tibial bowing. 

**Figure 2:**
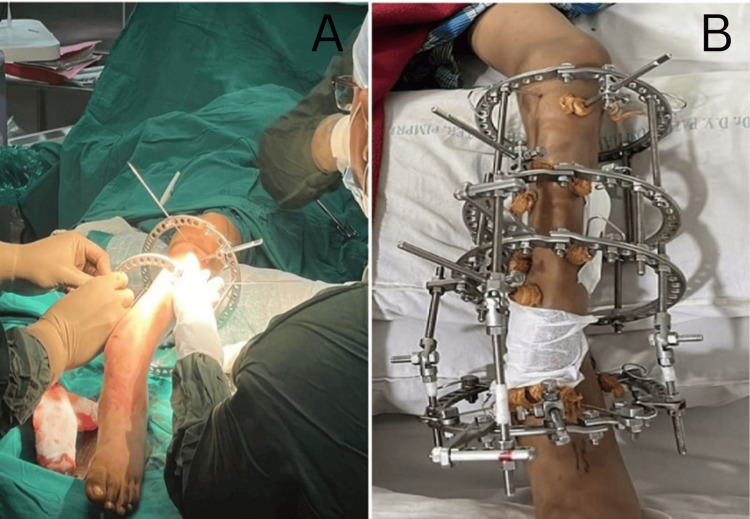
A) Intraoperative Ilizarov ring fixation and B) the post-operative clinical picture of Ilizarov fixator with pin tract dressing A) Intraoperative picture of pin insertions of Ilizarov external fixator and B) picture of Ilizarov assembly in place.

As the middle segment of CPT was long and sclerotic with a very narrow distal one-third calf due to fibular hemimelia with absent DPA, a plan of not doing an open procedure was followed. The proposed treatment plan involved corticotomy for deformity correction, followed by stabilization using Ilizarov’s ring fixation. The potential risks, including malunion, non-union, and the possibility of unsuccessful deformity correction, were discussed with the parents. Informed consent was subsequently obtained. The patient was positioned supine and underwent general anesthesia. Sterile preparation was meticulously performed, and a warmer was positioned at the head end to mitigate the risk of hypothermia. After painting and draping were done, the fracture was confirmed under a fluoroscope at the start of the procedure. Four Ilizarov rings, each 150 mm in diameter, were prepared on the trolley by the assistant surgeon at the start of the procedure based on the pre-operative measurements. Then the rings were attached to the tibia with the help of conical pins and k-wires. The rings were strategically positioned: the primary support ring proximally, a secondary support ring distally, and a pusher/puller ring approximately 7 cm distal to the fracture site along with the reference ring. These rings were interconnected using rods and hinges, with tension applied to the k-wires using a specialized tensioner. In this case, we applied the Ilizarov fixator in proximal tibia and distal fragment with hinge and motor construct across the fracture site to correct the mechanical axis. Gradual distraction followed by correction of angulation was done till normal mechanical axis was achieved. Fluoroscopic evaluation confirmed satisfactory fracture reduction. The surgical sites, including pin tracts, were dressed under sterile conditions (Figure 2). Distal peripheral pulses remained palpable, indicating an uneventful procedure.

In this case we applied the Ilizarov fixator in the proximal tibia and distal fragment with hinge and motor construct across the fracture site to correct the mechanical axis. Gradual distraction followed by correction of angulation was done till a normal mechanical axis was achieved. 

**Figure 3:**
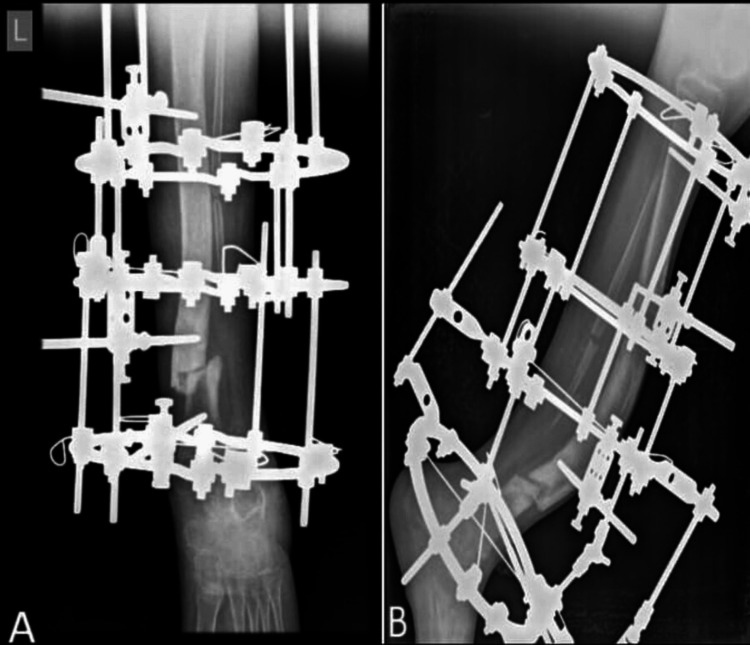
Post-operative X-rays showing Ilizarov fixation. A) Anteroposterior view; B) lateral view A) Two months post-operative X-ray (anteroposterior view) of the left tibia after correction of anterolateral bowing and compression at fracture site; B) two months post-operative X-ray (lateral view) of the left tibia after correction of anterolateral bowing and compression at the fracture site.

Post-operative radiological status of the patient was evaluated and presented in Figure 3. In the immediate post-operative phase, pain management was prioritized, and daily pin tract dressings were administered. Immediately following the surgery, the patient was started with ankle pump physiotherapy with gradual progression to bedside sitting over the course of a week. The principle of "distraction osteogenesis" was employed, in which bone and soft tissues were gradually distracted at a rate of 1 mm per day, divided into four increments. After a week, the patient began extending the injured limb, and pressure was kept up to maintain the force line stable. Emphasis was placed on non-weight-bearing ambulation during the initial post-operative week. Simultaneously, knee and ankle mobilizations were encouraged. In order to build strength, the knee and ankle were both mobilized at the same time, and the distance between the bones increased by 1 mm every day till the lengthening of 2 cm of the tibia was achieved along with the correction of the deformity. The patient was also fitted with heel raise shoes and commenced weight-bearing ambulation with the aid of a walker. The pre-operative center of rotation of angulation (CORA) was 60° which showed an improvement to 25°, post-operatively.

**Figure 4:**
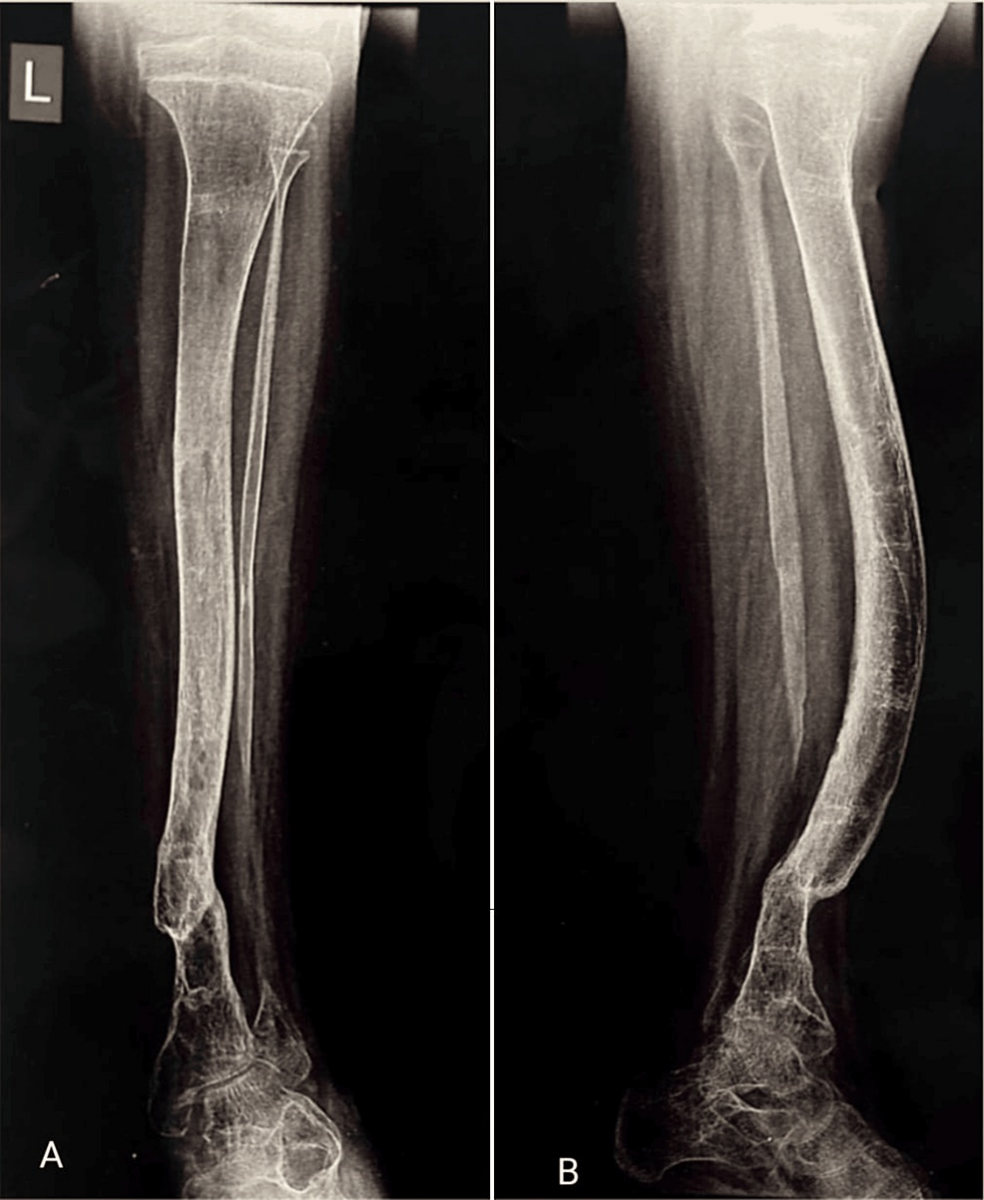
One year post-operative X-ray of left tibia showing the union of fracture. A) Anteroposterior view; B) lateral view A) One year post-operative X-ray (anteroposterior view) of the left tibia showing the union of the fracture site and B) one year post-operative X-ray (lateral view) of the left tibia showing the union of fracture site.

Monthly follow-ups, inclusive of radiographic evaluations, were scheduled for the first six months. Seven months after the surgery, the external fixator was removed. Since the fracture union was unstable, the patient began practicing partial weight-bearing activities with the use of a specialized brace, a month after it was removed. He avoided vigorous activity for the nearby joints and made the transition to full weight-bearing ambulation gradually over one year (Figure 4).

## Discussion

CPT is one of the challenging orthopedic conditions, with an ambiguous pathophysiology and varied outcomes following the therapy. Every therapeutic approach seeks to achieve a sustained bony fusion of the tibia and fibula, mitigate limb-length differences, prevent deviations in the mechanical axis, protect against soft tissue injuries, maintain nearby joint mobility, and avert pathological fractures [5,6]. The index case of CPT with anterolateral bowing that led to a stress fracture of the tibia due to cyclic loading of bone is a rare report from India. In this case, the child was doing full weight bearing, walking with deformity before he had a fracture, following which he stopped walking. Axis deviation with bowing and poor bone quality might have led to the pathological stress fracture. Because the early symptoms are so similar to those of fractures, CPT is often misdiagnosed [7].

The bone loss was zero, as no tibial osteotomy was done in the management, and we tried to fix the deformity by stabilizing the fracture using Ilizarov’s ring fixator. Ilizarov’s ring fixation technique has been an efficient therapeutic option for CPT [7]. Concurrent fixation of the fibula is recommended since a fibular non-union may lead to valgus deformities [1]. A combination of Ilizarov’s fixation with “Eiffel Tower” double titanium elastic nails (TENs) has also been shown to improve bone union and functional outcomes among CPT patients [8]. A recent case series from Egypt showed that Ilizaraov’s distraction in combination with the intramedullary fibular graft prevented osteotomy of the tibia. It also aided in correcting the limb-length shortening [9]. A systematic review revealed that the combination therapy produced better outcomes than the techniques (intramedullary rod or Ilizarov) used in solitary [10]. There was no bone graft applied in the index case, which could have been used as a form of a simple graft or an inter-tibia-fibular graft. It may be performed during the same procedure or in a second step to reinforce the union. No complications were observed in the index case during our follow-up duration of eight months after the procedure. However, longer-duration follow-ups were done in other studies (up to six years, until skeletal maturity) and reported varied complications and functional outcomes [11,12].

CPT has a strong association with NF1 (up to 80%) [13]. The index case was a known patient of NF1, and usually, the diagnosis of the CPT precedes the NF1 manifestations [14]. The close association between NF1 and the CPT should be leveraged to undertake genetic testing and counseling approach to assess for NF1 among CPT patients to enable earlier diagnosis of NF1 [14]. The management of the index case was challenging since the CPT had convex anterolateral bowing associated with aneuro-fibromatosis, fibular hemimelia, and poor skin conditions. The relatively older age (12 years) at which the index case presented was another major risk factor for poor outcomes. The younger age of the patients (mean age: 2.37 years) at the time of the first surgery and the distal location of the CPT enable favorable outcomes [15].

## Conclusions

Ilizarov procedure is one of the effective therapeutic options for CPT patients. Multiple treatment outcomes such as osteosynthesis, limb-length lengthening, and ankle stabilization can be achieved by this technique simultaneously. Correction of the mechanical axis helps in achieving union in this difficult case. The patient needs to be followed up till he achieves skeletal maturity to understand the functional outcomes of the procedure, comprehensively.
